# On the Cure of Tonsillitis by Mercury

**Published:** 1830-10-01

**Authors:** 


### ON THE CURE OF TONSILLITIS BY MEANS OF CALOMEL By Dr. BUET.

[Journ. Complement.]

THE French are beginning to shake off their hydrargyro-phobia, and we should not be much surprised if they give mercury more freely than their English neighbours in the course of a few years.

Dr. Buet has lately drawn the attention of his countrymen to the exhibition of calomel both in acute and chronic amygdalitis, informing them that this plan is by far the most efficacious in the reduction of those chronic enlargements of the tonsils, which embarrass deglutition and alter the voice so much as sometimes to render the excision of the glands necessary. In the Journal Hebdomad. for May, 1830, M. Mondezert published some cases treated on the mercurial plan, and the recital of these cases drew M. Buet's attention to the subject. Six cases are detailed by the latter physician, some of which we shall briefly notice.

The first case was of a chronic nature; and the difficulty of deglutition had been troublesome for four months. The tonsils were enlarged and indurated. Six grains of calomel and thirty grains of powdered savine leaves were divided into six doses, and one of these was ordered night and morning. The pain and sense of difficulty in deglution were, at first, increased; but soon diminished, and in a week the patient was cured.

In three cases of acute amygdalitis, Dr. Buet observed the most striking advantages to result from the administration of calomel. The first of these was a female who experienced a rigor, and then a most violent inflammation of the tonsils and throat generally. She would not submit either to leeches or venesection. Calomel in the manner above-mentioned, was therefore prescribed; and, on the third day, the patient was able to resume her avocations. The other cases had a similar result.

We do not attach very much importance to this measure; but we think it is worthy of remembrance in chronic cases, where, however, we would recommend the auxiliary assistance of iodine, either in the shape of frictions with the hydriodate of potash, or the tincture of iodine, beginning with doses of four drops thrice a day, and gradually increasing the medicine to 20 drops per die.

---

